# Educational differences in alcohol-related morbidity and the role of working conditions: a Swedish register-based cohort study

**DOI:** 10.1093/eurpub/ckae158

**Published:** 2024-10-17

**Authors:** Melody Almroth, Tomas Hemmingsson, Daniel Falkstedt, Emma Carlsson, Katarina Kjellberg, Emelie Thern

**Affiliations:** Institute of Environmental Medicine, Karolinska Institutet, Stockholm, Sweden; Department of Public Health Sciences, Stockholm University, Stockholm, Sweden; Institute of Environmental Medicine, Karolinska Institutet, Stockholm, Sweden; Department of Public Health Sciences, Stockholm University, Stockholm, Sweden; Institute of Environmental Medicine, Karolinska Institutet, Stockholm, Sweden; Department of Public Health Sciences, Stockholm University, Stockholm, Sweden; Department of Global Public Health, Karolinska Institutet, Stockholm, Sweden; Institute of Environmental Medicine, Karolinska Institutet, Stockholm, Sweden; Centre for Occupational and Environmental Medicine, Region Stockholm, Stockholm, Sweden; Institute of Environmental Medicine, Karolinska Institutet, Stockholm, Sweden; Department of Public Health Sciences, Stockholm University, Stockholm, Sweden

## Abstract

This study aims to investigate the relationship between education and alcohol-related morbidity and the role that low job control and heavy physical workload play in explaining these associations among men and women in Sweden. This register-based cohort study (SWIP cohort) includes over three million individuals registered in Sweden in 2005. Job control and physical workload were measured using a job exposure matrix linked to the index person based on their registered occupation at baseline. Alcohol-related morbidity was measured through diagnoses in the national patient registers between 2006 and 2020. Cox proportional hazards regression models were built to estimate associations between education and alcohol-related morbidity. Reductions in hazard ratios (HRs) were calculated after adjusting for job control, physical workload, and other covariates. Models were also stratified by sex. Lower levels of education predicted a higher risk of alcohol-related morbidity (HR: 2.55 95% confidence interval: 2.49–2.62 for the lowest educated compared to the highest). Low job control and heavy physical workload both played roles in explaining educational differences in alcohol-related morbidity even after accounting for sociodemographic and health factors (15.1% attenuation for job control and 18.3% for physical workload among the lowest educated). Physical workload explained a larger proportion of the associations among men compared to women. Lower levels of education were associated with an increased risk of alcohol-related morbidity and working conditions partly explained these associations beyond what was explained by sociodemographic and health factors. Improving working conditions could therefore prevent some cases of alcohol-related morbidity.

## Introduction

Alcohol-related morbidity is common, accounting for around 5% of the global burden of disease [1], but tends to be unequally distributed in the population. Those with lower socioeconomic status and lower education are at greater risk for alcohol-related harm [2–4]. Even when genetic factors and alcohol consumption patterns are accounted for, alcohol-related consequences tend to be higher among lower socioeconomic groups [5, 6]. The explanation of these patterns is of great interest but not fully understood. Most previous studies have only focused on behavioral explanations at the individual level [7], and more research on societal and contextual root causes of social inequalities in alcohol-related harm has been called for [8].

Working conditions may be an important determinant of socioeconomic inequalities in alcohol-related morbidity and have previously been found to partially explain educational inequalities in health behaviors [9]. Those with lower levels of education are more likely to work in occupations where they are exposed to poor working conditions [10, 11]. Poor working conditions can, in turn, increase the risk of alcohol-related morbidity [12]. Individuals may drink to relieve tension from psychosocial stress [13, 14]. Alcohol may also be used to cope with fatigue from physically exhausting work [13, 15], or as pain relief [16]. A lack of stimulation or challenge at work could also lead to higher alcohol consumption due to coping with boredom [17].

Several previous studies including a meta-analysis and systematic review [18, 19] have reported associations between psychosocial workplace factors and various measures of risky or hazardous alcohol use or alcohol-related morbidity [12, 18–24]. One study from our own group showed that low job control is associated with alcohol-related morbidity [25]. Some studies have found that physical exertion at work is related to negative alcohol-related outcomes [13, 15, 26, 27], though this phenomenon has less frequently been investigated. No known studies have looked at the explanatory role of work environment exposures in the relationship between education and alcohol-related harm.

Differences between men and women are also important to consider as men and women tend to hold different occupations and positions and may have different exposures within the same occupations. Sweden has a more segregated labor market than the European average [28]. Women and men also have different patterns in terms of alcohol consumption where men report higher consumption [29]. It has been suggested that women may be more vulnerable to harmful effects of alcohol even though they have a lower absolute disease burden [5]. Thus, it is important to consider sex-specific patterns in educational differences in alcohol-related morbidity and the role of working conditions.

The aim of this study is to investigate the relationship between education and alcohol-related morbidity and the role that low job control and heavy physical workload play in explaining these associations among men and women in Sweden. This study will contribute to a better understanding of potential mechanisms explaining educational inequalities in alcohol-related morbidity, which are still largely unknown.

## Methods

### Study population and design

This study uses the SWIP cohort (Swedish Work, Illness, and labor-market Participation) which includes all individuals between the ages of 16 and 64 who were registered as living in Sweden during the year 2005 (around 5.4 million). National register data are linked from the major administrative and health registers in Sweden. This includes the total population register [30], the LISA register (Longitudinal Integrated Database for Health Insurance and Labor Market Studies) [31], and the national patient register which includes mandatory reported information from inpatient and specialist care. Index persons are also linked to their parents’ census information from 1960, 1970, and 1980. The SWIP cohort has previously been described in other publications [32, 33].

For this study, we restricted the study population to those who were 30–60 years old in 2005 (born 1945–1975) to focus on those who were more likely to be established and remaining in the labor market at baseline. This resulted in a population of around 3.8 million individuals. We excluded those who did not have occupational information in 2005 (741 555). We further excluded those with a previous alcohol-related diagnosis prior to 2005 (40 605), and those with missing information on any covariate (6491). After all exclusions, our final sample was 3 017 052 individuals (51% women).

Ethical approval for the present study was obtained by the Regional Ethics Review Board in Stockholm, reference numbers 2017/1224-31 and 2018/1675-32 and 2022/02725-02.

### Measures

*The highest attained education* was measured from the LISA register in 2005 and categorized into five categories. This included primary and lower secondary school (≤9 years), secondary school (10–11 years), upper secondary school (12 years), post-secondary/university—2 years or less (13–14 years) and at least three years of post-secondary/university education (≥15 years).

*Alcohol-related morbidity* was measured based on the national inpatient and outpatient registers from 2006 to 2020. The Swedish version of the International Classification of Diseases, version 10 (ICD-10); codes F10, K70, and T51 were used for the inpatient register and the same codes with the addition of E24.4, G31.2, G62.1, G72.1, I42.6, K29.2, K85.2, K86.0, O35.4, Y90, Y91, Z50.2, Z71.4, Z72.1 [34] were used in the outpatient register due to the availability of more specific diagnostic information. Supplementary Table S1 shows the definitions of each diagnosis. Cases were considered as those with one of these diagnoses as the main or contributing diagnosis in either of the patient registers during follow-up.

*Job control* was measured using a job exposure matrix (JEM) which is based on around 90 000 responses to the Swedish Work Environment Surveys. These surveys were answered every second year in the period of 1997–2013. Survey responses are aggregated on the occupational level based on 355 occupations for men and women separately. These exposure levels are then linked to the index person based on their registered occupation during 2005. This four-digit occupational code is based on the Swedish version of the International Classification of Occupations (ISCO-88).

The specific measure for job control was estimated using the average answers to four questions measuring decision authority and three questions measuring skill discretion. The decision authority questions measure the ability of the person to determine which tasks to do, the pace of their work, when they can take breaks, and the structure of their work [32]. The questions regarding skill discretion measure opportunities for learning and development, problem solving, and training opportunities [35]. Because the JEM itself is sex-specific, and in order to reflect a range of exposure levels in the population, job control was categorized into sex-specific quintiles based on the distribution in the study population, which is in line with previous studies [25, 32].

*Physical workload* was also measured using a JEM based on the same Swedish Work Environment Surveys. This was measured using an index of eight survey items regarding physical work that involves heavy lifting, uncomfortable working postures, repetitive work, and physically demanding work [33]. After linkage to the index person through their occupational code, physical workload was also categorized into sex-specific quintiles based on the population distribution.

*Age, sex, and country of birth* were measured from the total population register. Country of birth was categorized to indicate Sweden or other.

*Childhood socioeconomic position* (SEP) was measured by linking index persons to their parents’ Swedish National Population and Housing Census information from when the index person was between 5 and 15 years old. This information comes from 1960, 1970, or 1980. Occupational information was categorized as unskilled manual workers, skilled manual workers, assistant non-manual workers, intermediate non-manual workers, professional non-manual workers, farmers, and those with no parental occupation reported. The father’s occupation was primarily used, but the mother’s was used if this information was missing.

*Parents’ previous psychiatric diagnoses* were taken from the inpatient register prior to baseline. If one of the parents had any ICD-10 diagnosis in the F chapter or an equivalent diagnosis in earlier versions of the ICD, and this diagnosis was given before the parent reached the age of 65, the index person would be classified as having a parent with a previous psychiatric diagnosis. Psychiatric diagnoses received after the age of 65 may be less relevant for genetic factors and social situations experienced in childhood.

*Index persons’ previous psychiatric diagnoses* were similarly measured using inpatient information prior to baseline. Any diagnosis in the ICD-10 F chapter or equivalent was used to identify those with previous psychiatric diagnoses. Those with previous alcohol-related morbidity as identified above were excluded.

*Marital status* was obtained from the total population register in 2005. The possible categories were unmarried, married, divorced, or widowed. *The number of children* was obtained from the LISA register which indicates how many children under the age of 20 are living in the household. We categorized this as 0, 1–2, or 3 or more children.

### Statistical analysis

The distribution of all covariates according to level of education was estimated. We also estimated the association between each covariate separately and alcohol-related morbidity using Cox proportional hazards regression with age as the underlying time scale in all models.

To estimate associations between education and alcohol-related morbidity and the explanatory role of working conditions, we used Cox regression models. Follow-up time started from 1 January 2006 and ended in the event of an alcohol-related diagnosis, the end of follow-up (31 December 2020), death, or migration from Sweden.

In the crude model, we regressed alcohol-related morbidity on level of education. In Model 1, we added the sociodemographic and background factors of birthyear, birth country, childhood SEP, parents’ psychiatric diagnoses, own psychiatric diagnoses, marital status, and number of children. In Model 2, we adjusted for all covariates in Model 1, plus job control in 2005. In Model 3, we adjusted for all covariates in Model 1 plus physical workload in 2005. In Model 4, we adjusted for all covariates in Model 1 plus both job control and physical workload.

Percent attenuation in the hazard ratios (HRs) for the educational groups after the inclusion of each set of covariates was calculated to determine the extent that associations were explained by including these factors. The percentage of HR reduction was calculated using the formula ((HR crude–HR adjusted)/(HRcrude–1)) × 100 where Model 1 was first compared to the crude model, and all subsequent models were compared to Model 1. Ninety-five percent confidence intervals (CIs) for the percentage of HR attenuation were obtained through bootstrap estimation using Efron’s quantile method performed with 100 resamplings.

To investigate potential sex differences, analyses estimating educational differences in alcohol-related morbidity and the explanatory role of working conditions were repeated for men and women separately. An interaction term between education and sex was also added to the models.

All analyses were done using SAS Enterprise Guide 8.3.

## Results

During the follow-up period, 49 852 men (3.3%) and 26 211 (1.7%) women received alcohol-related diagnoses from in- or outpatient care. The average follow-up time was 14.38 years with a total of 43 405 865.6 person-years.

Those with lower levels of education tended to be slightly older, were more often men, more likely to be born outside of Sweden, to have parents from lower socioeconomic classes as well as parents more likely to have had a previous psychiatric diagnosis, to have had a previous psychiatric diagnosis themselves, to be divorced or widowed, and to not have children living at home (Table 1). Low job control and heavy physical workload were also much more common among the lower educated, as was receiving an alcohol-related diagnosis during follow-up.

**Table 1.** Covariates according to the level of education

	≥15		13–14		12		10–11		≤9	
	*N*	%	*N*	%	*N*	%	*N*	%	*N*	%
Total	660 906	21.9	477 057	15.8	479 176	15.9	1 002 198	33.2	397 715	13.6
Age (mean, SD)	43.5	9.2	44.5	8.5	42.6	9.3	45.2	8.4	48.6	8.5
Female sex	369 766	55.9	256 751	53.8	244 390	51.0	494 649	49.4	162 410	40.8
Born outside Sweden	86 710	13.1	49 928	10.5	60 046	12.5	100 781	10.1	65 972	16.6
Childhood SEP										
Unskilled manual	102 011	15.4	102 255	21.4	123 489	25.8	332 757	33.2	137 050	34.5
Skilled manual	90 817	13.7	89 834	18.8	102 477	21.4	253 785	25.3	91 131	22.9
Lower non-manual	79 120	12.0	59 138	12.4	55 756	11.6	89 424	8.9	26 026	6.5
Intermediate non-manual	177 085	26.8	110 794	23.2	87 429	18.2	122 658	12.2	29 875	7.5
Professional non-manual	91 200	13.8	35 591	7.5	23 864	5	22 657	2.3	6261	1.6
Farmer	28 432	4.3	25 013	5.2	22 163	4.6	62 139	6.2	30 848	7.8
Not classified	92 241	14.0	54 432	11.4	63 998	13.4	118 778	11.9	76 524	19.2
Parents previous psych	35 307	5.3	27 354	5.7	34 206	7.1	70 089	7.0	24 199	6.1
Own previous psych	18 984	2.9	15 080	3.2	15 122	3.2	40 813	4.1	17 464	4.4
Marital status										
Unmarried	213 286	32.3	153 293	32.1	185 066	38.6	356 473	35.6	119 984	30.2
Married	374 216	56.6	261 576	54.8	234 438	48.9	491 358	49.0	206 675	52.0
Divorced	68 709	10.4	58 331	12.2	56 169	11.7	143 556	14.3	64 680	16.3
Widowed	4695	0.7	3857	0.8	3503	0.7	10 811	1.1	6376	1.6
Number of children										
0	294 341	44.5	212 269	44.5	217 497	45.4	490 904	49.0	245 261	61.7
1–2	303 954	46.0	217 999	45.7	216 733	45.2	413 623	41.3	122 245	30.7
≥3	62 611	9.5	46 789	9.8	44 946	9.4	97 671	9.7	30 209	7.6
Job control 2005										
Low	22 221	3.4	42 480	8.9	88 167	18.4	245 649	24.5	158 084	39.7
Medium low	69 596	10.5	63 366	13.3	109 728	22.9	287 913	28.7	95 464	24.0
Medium	145 373	22.0	119 997	25.2	72 683	15.2	190 961	19.1	61 056	15.4
Medium high	121 423	18.4	115 870	24.3	136 112	28.4	201 913	20.1	59 926	15.1
High	302 293	45.7	135 344	28.4	72 486	15.1	75 762	7.6	23 185	5.8
Physical work 2005										
Low	319 126	48.3	123 193	25.8	71 806	15.0	71 441	7.1	20 402	5.1
Medium low	176 160	26.7	116 087	24.3	118 718	24.8	152 635	15.2	43 172	10.9
Medium	117 406	17.8	159 381	33.4	89 304	18.6	171 727	17.1	62 044	15.6
Medium high	29 161	4.4	44 416	9.3	98 561	20.6	296 484	29.6	134 446	33.8
High	19 053	2.9	33 980	7.1	100 787	21.0	309 911	30.9	137 651	34.6
Alcohol-related morbidity	9900	1.5	9103	1.9	11 357	2.4	30 395	3.0	15 308	3.9

≥15 = three or more years of university; 13–14 = <3 years of university; 12 = 3 years of upper secondary school; 10–11 = <3 years of upper secondary school; ≤9 = compulsory school or less.

SEP = socioeconomic position; psych = psychiatric diagnosis.

Age, lower childhood SEP, the presence of parents’ and own psychiatric diagnoses, and being unmarried, divorced, or widowed were all associated with an increased risk of receiving an alcohol-related diagnosis during follow-up (Table 2). Women and those living with children had a lower risk of developing an alcohol-related illness. Lower job control and higher physical workload were associated with an increased risk of receiving an alcohol-related diagnosis, and this showed a dose–response pattern for physical workload.

**Table 2.** Hazard ratios and 95% confidence intervals of covariates according to alcohol-related morbidity among women and men

	HR (95% CI)
Age	1.01 (1.01–1.01)
Female sex	0.50 (0.49–0.51)
Born outside of Sweden	0.95 (0.93–0.97)
Childhood SEP	
Unskilled manual	1.23 (1.19–1.28)
Skilled manual	1.19 (1.15–1.23)
Lower non-manual	1.20 (1.16–1.25)
Intermediate non-manual	1.06 (1.03–1.10)
Professional non-manual	1.00
Farmer	0.57 (0.54–0.60)
Not classified	1.10 (1.06–1.14)
Parents’ previous psych diagnosis	1.78 (1.74–1.82)
Own previous psych diagnosis	3.83 (3.74–3.91)
Marital status	
Unmarried	1.75 (1.72–1.78)
Married	1.00
Divorced	2.29 (2.24–2.33)
Widowed	1.59 (1.49–1.70)
Number of children	
0	1.00
1–2	0.60 (0.59–0.61)
≥3	0.59 (0.57–0.61)
Job control 2005	
Low	1.79 (1.74–1.83)
Medium low	1.81 (1.76–1.85)
Medium	1.39 (1.35–1.42)
Medium high	1.29 (1.26–1.33)
High	1.00
Physical workload 2005	
Low	1.00
Medium low	1.12 (1.09–1.15)
Medium	1.47 (1.44–1.51)
Medium high	1.62 (1.58–1.66)
High	1.75 (1.71–1.79)

SEP = socioeconomic position; Psych = psychiatric.

A clear gradient was found where the lower the education level, the greater the risk of receiving an alcohol-related diagnosis (HR: 1.25 95% CI: 1.22–1.29 for those with 13–14 years of education and HR: 2.55 95% CI: 2.49–2.62 for those with 9 years or fewer compared to those with at least 15 years of education) (Table 3). Adjusting for the sociodemographic characteristics of sex, birthyear, birth country, childhood SEP, parents’ history of psychiatric diagnoses, own history of psychiatric diagnoses, marital status, and number of children living at home attenuated the associations between education and alcohol-related morbidity by 26.7% (95% CI: 25.8–27.4) for the lowest educated group (Model 1). Additionally adjusting for job control further attenuated associations by 15.1% (95% CI: 13.6–16.2) beyond what was explained by the covariates in Model 1 in the lowest educated group (Model 2). Adjusting for physical workload in addition to the sociodemographic characteristics led to a reduction of 18.3% (95% CI: 16.7–20.2) beyond what was explained by the covariates in Model 1 among the lowest educated (Model 3). Finally, including both job control and physical workload in addition to the sociodemographic covariates led to a similar reduction of 17.4% (95% CI: 15.8–19.3) in the lowest educated group.

**Table 3.** Crude and adjusted hazard ratios (HRs) and 95% confidence intervals (CIs) for associations between education level and alcohol-related morbidity

	>15	13–14		12		10–11		<9	
	HR	HR (95% CI)	%Δ (95% CI)	HR (95% CI)	%Δ (95% CI)	HR (95% CI)	%Δ (95% CI)	HR (95% CI)	%Δ (95% CI)
Crude	1.00	1.25 (1.22–1.29)		1.58 (1.54–1.62)		1.99 (1.94–2.03)		2.55 (2.49–2.62)	
Model 1	1.00	1.21 (1.17–1.24)	17.2 (15.0–19.3)	1.49 (1.45–1.53)	15.8 (14.6–16.6)	1.78 (1.74–1.82)	21.0 (20.1–21.9)	2.14 (2.08–2.20)	26.7 (25.8–27.4)
Model 2	1.00	1.17 (1.14–1.21)	15.7 (13.3–18.5)	1.41 (1.37–1.45)	16.6 (14.8–18.3)	1.64 (1.60–1.69)	17.6 (15.9–18.8)	1.97 (1.91–2.02)	15.1 (13.6–16.2)
Model 3	1.00	1.16 (1.13–1.20)	21.7 (18.8–25.2)	1.40 (1.36–1.44)	18.0 (16.4–20.2)	1.62 (1.58–1.67)	20.1 (18.4–22.2)	1.93 (1.87–1.99)	18.3 (16.7–20.2)
Model 4	1.00	1.17 (1.13–1.20)	18.7 (16.1–21.9)	1.40 (1.36–1.44)	18.6 (17.1–20.6)	1.62 (1.58–1.67)	20.2 (18.4–22.3)	1.94 (1.88–2.00)	17.4 (15.8–19.3)

Model 1 is adjusted for sex, birth year, birth country, childhood socioeconomic position, parents’ psychiatric diagnoses, own previous psychiatric diagnoses, marital status, and number of children.

Model 2 is adjusted for covariates in Model 1 + job control in 2005.

Model 3 is adjusted for covariates in Model 1 + physical workload in 2005.

Model 4 is adjusted for covariates in Model 1 + physical workload and job control in 2005.

%Δ percent reduction in hazard ratio using the formula ((HR1–HR2)/(HR1–1)) × 100 where Model 1 is compared to the crude model and all other models are compared to Model 1.

Interaction terms between sex and education were not significant, but sex-stratified analyses showed that physical workload led to greater HR attenuations among men compared to women (10.4%, 95% CI: 7.1–13.2 for women and 22.7%, 95% CI: 20.8–24.6 for men) (Table 4).

**Table 4.** Crude and adjusted hazard ratios (HRs) and 95% confidence intervals (CIs) for associations between education level and alcohol-related morbidity stratified by sex

	≥15	13–14		12		10–11		≤9	
	HR	HR (95% CI)	%Δ (95% CI)	HR (95% CI)	%Δ (95% CI)	HR (95% CI)	%Δ (95% CI)	HR (95% CI)	%Δ (95% CI)
Women									
Crude	1.00	1.25 (1.19–1.30)		1.46 (1.40–1.53)		1.92 (1.86–2.00)		2.36 (2.26–2.47)	
Model 1	1.00	1.23 (1.17–1.29)	7.5 (5.2–10.6)	1.43 (1.37–1.50)	6.4 (4.6–8.2)	1.82 (1.75–1.89)	11.7 (10.1–13.2)	2.19 (2.09–2.29)	12.9 (11.3–14.5)
Model 2	1.00	1.21 (1.15–1.26)	10.0 (7.4–12.4)	1.33 (1.27–1.40)	22.6 (19.1–26.3)	1.66 (1.60–1.73)	18.9 (16.5–21.0)	2.02 (1.92–2.12)	14.3 (11.8–15.7)
Model 3	1.00	1.19 (1.14–1.25)	15.4 (11.3–20.3)	1.39 (1.32–1.45)	10.4 (6.7–13.7)	1.72 (1.65–1.80)	11.2 (7.7–14.4)	2.06 (1.96–2.17)	10.4 (7.1–13.2)
Model 4	1.00	1.20 (1.15–1.26)	12.3 (8.4–17.4)	1.35 (1.29–1.42)	18.0 (14.4–21.7)	1.69 (1.62–1.76)	15.8 (12.7–19.1)	2.04 (1.94–2.15)	12.1 (8.9–15.0)
Men									
Crude	1.00	1.23 (1.19–1.28)		1.56 (1.50–1.61)		1.93 (1.88–1.99)		2.30 (2.22–2.37)	
Model 1	1.00	1.21 (1.17–1.25)	10.8 (11.9–14.6)	1.49 (1.44–1.55)	11.3 (9.8–12.6)	1.81 (1.76–1.87)	13.1 (11.3–14.3)	2.12 (2.05–2.19)	13.5 (11.9–14.6)
Model 2	1.00	1.17 (1.13–1.22)	16.8 (13.7–20.4)	1.41 (1.36–1.46)	17.3 (15.1–19.6)	1.66 (1.60–1.71)	19.0 (17.2–21.2)	1.92 (1.86–1.99)	17.6 (15.8.19.7)
Model 3	1.00	1.16 (1.12–1.20)	23.5 (20.0–28.1)	1.38 (1.33–1.43)	22.5 (19.8–25.3)	1.61 (1.56–1.66)	24.8 (22.3–27.2)	1.87 (1.80–1.94)	22.7 (20.8–24.6)
Model 4	1.00	1.17 (1.12–1.21)	20.3 (16.5–24.4)	1.39 (1.34–1.44)	20.5 (17.5–23.0)	1.62 (1.57–1.68)	23.0 (20.1–25.4)	1.88 (1.82–1.96)	21.2 (18.6–23.2)

Model 1 is adjusted for birth year, birth country, childhood socioeconomic position, parents’ psychiatric diagnoses, own previous psychiatric diagnoses, marital status, and number of children.

Model 2 is adjusted for covariates in Model 1 + job control in 2005.

Model 3 is adjusted for covariates in Model 1 + physical workload in 2005.

Model 4 is adjusted for covariates in Model 1 + physical workload and job control in 2005.

%Δ percent reduction in hazard ratio using the formula ((HR1–HR2)/(HR1–1)) × 100 where Model 1 is compared to the crude model and all other models are compared to Model 1.

## Discussion

This study found clear associations between lower levels of education and higher risks for alcohol-related morbidity and that low job control and heavy physical workload both played roles in explaining these associations among men and women even after accounting for a variety of sociodemographic and health factors. Physical workload explained a greater proportion of associations among men compared to women.

That those with lower education had a higher risk of alcohol-related morbidity is in line with previous studies showing a similar pattern with, for example, alcohol use disorder and dependency [3, 5].

Our results supported the hypothesis that job control as well as physical workload played explanatory roles in educational differences in alcohol-related morbidity for both men and women. We are unaware of any previous study which looked at working conditions as explanatory factors in educational differences in alcohol-related morbidity, and thus make a contribution to the field in this regard.

Some previous studies, however, have looked at associations between working conditions and alcohol-related outcomes. Similar to our own findings, two Swedish studies found that low job control was related to increased risks of alcohol-related harm [21, 25]. One Norwegian study found low job control to predict alcohol use after the workday, but not problematic alcohol use [22]. A Finnish study did not find any associations between job control and heavy drinking for men or women [12]. These latter two studies were based on questionnaire data with self-reported working conditions and alcohol consumption in contrast to the present study which used a JEM and alcohol-related diagnoses. Using the same informant to estimate both working conditions and alcohol consumption could lead to bias, as reporting biases might not be independent of each other.

Also similar to the present study, several previous studies have investigated physical workload in relation to alcohol-related outcomes. These studies, however, only found higher physical workload to be related to alcohol-related outcomes among men [13, 15, 26, 27]. Most of these studies also relied on self-reported data only. We found, contrarily, that physical workload predicted alcohol-related morbidity in both men and women.

We did not observe sex differences in the relationship between education and alcohol-related morbidity but found that physical workload explained more of the associations among men. A meta-analysis of socioeconomic differences in alcohol-related mortality found similar patterns among men and women when education was used as the measure of socioeconomic differences but found stronger associations among men when occupation was used as the measure of socioeconomic differences [36]. Physically heavy jobs are more common among men, and there could be gender differences in workplace culture and acceptability of alcohol use which are concentrated in more physical jobs among men.

Both low job control and heavy physical workload explained some of the educational differences in alcohol-related morbidity may be explained through coping and self-medication mechanisms [13]. These mechanisms may link poor psychosocial and physical work environments, which are more common among the lower educated, to an increased risk of alcohol-related harm. There may be additional work-related aspects which are partially captured through low job control and heavy physical workload that could also explain differences in alcohol-related morbidity. An example could be higher social acceptability for drinking which was recently found to predict problematic drinking in a Norwegian study [37] and which could be more common among lower control and higher physical jobs.

### Strengths and limitations

A strength of this study is the register-based design which represents the entire registered Swedish population and decreases issues of selection and attrition. Another strength is the possibility to assess alcohol-related diagnoses continuously and to be able to exclude those with previous alcohol-related diagnoses which occurred before follow-up. This is useful for assessing the temporality of the relationship between education, working conditions, and alcohol-related morbidity. Additionally, we were able to adjust for a variety of potentially confounding factors thought to be relevant to the exposure–outcome relationship. This included factors from childhood, as well as factors representing health, and family situation.

This study also had some limitations. While we were able to control for several relevant covariates, we were not able to control for genetic factors potentially associated with both an increased risk of alcohol-related morbidity and lower SEP. Additionally, we were not able to adjust for health behavioral factors including diet, physical activity, smoking, and most importantly, previous alcohol consumption. Our use of JEMs for measuring working conditions allows for the possibility to study the entire population and reduces issues of common methods bias but leads to inevitable misclassification because the exposure is measured at the occupational rather than individual level. Since exposures are measured at the occupational level, they are highly correlated, making it difficult to disentangle their separate effects. Relying on patient registers for alcohol-related morbidity captures only the more severe treated cases. We chose a more inclusive definition of alcohol-related morbidity that represents a wide range of different diagnoses, but there may be different patterns according to specific diagnoses which were outside the scope of this study.

## Conclusion

Those with lower education are at a greater risk of developing alcohol-related morbidity. Low job control and heavy physical workload are supported as mechanisms which may partially explain the association between education and alcohol-related morbidity among men and women. These factors appear important beyond what is explained by sociodemographic and health vulnerabilities. Improving working conditions could therefore prevent some cases of alcohol-related morbidity.

## Supplementary Material

ckae158_Supplementary_Data

## Data Availability

The data used in this analysis are based on national register data held by Statistics Sweden (SCB). Key points No known previous studies have investigated working conditions as a mechanism for explaining educational differences in alcohol-related morbidity. This study found that low job control and heavy physical workload play a role in explaining the relationship between education and alcohol-related morbidity beyond what is explained by sociodemographic and health factors. Improving working conditions in terms of job control and physical workload could improve inequalities in alcohol-related morbidity.
