# Supplementary material for: Educational differences in alcohol-related morbidity and the role of working conditions: a Swedish register-based cohort study
Source: Eur J Public Health. 2024 Oct 17;34(6):1134–9. doi: 10.1093/eurpub/ckae158 (PMC11631502; doi:10.1093/eurpub/ckae158)
Supplement: ckae158_Supplementary_Data [file ckae158_supplementary_data.pdf]

**Table S1** ICD-10 codes and descriptions

| ICD-10 code | Definition |
|---|---|
| E24.4 | alcohol-induced pseudo-Cushing's syndrome |
| F10 | mental and behavioral disorders due to alcohol |
| G31.2 | degeneration of nervous system due to alcohol |
| G62.1 | alcoholic polyneuropathy |
| G72.1 | alcoholic myopathy |
| I42.6 | alcoholic cardiomyopathy |
| K29.2 | alcoholic gastritis |
| K70 | alcoholic liver disease |
| K85.2 | alcohol induced acute pancreatitis |
| K86.0 | alcohol induced chronic pancreatitis |
| O35.4 | maternal care for (suspected) damage to fetus from alcohol |
| R780 | finding of alcohol in blood |
| T51 | toxic effect of alcohol |
| Z50.2 | rehabilitation due to alcohol |
| Z71.4 | alcohol abuse counseling and surveillance |
| Z72.1 | lifestyle problems due to alcohol |
